# Animal bioturbation preserved in Pleistocene magadiite at Lake Magadi, Kenya Rift Valley, and its implications for the depositional environment of bedded magadiite

**DOI:** 10.1038/s41598-020-63505-7

**Published:** 2020-04-22

**Authors:** Luis A. Buatois, Robin W. Renaut, Richard Bernhart Owen, Anna K. Behrensmeyer, Jennifer J. Scott

**Affiliations:** 1 Department of Geological Sciences, University of Saskatchewan, 114 Science Place, Saskatoon, SK S7N 5E2 Canada; 2 Department of Geography, Hong Kong Baptist University, Kowloon Tong, Hong Kong; 3 Department of Paleobiology, Smithsonian Institution, Washington DC, 20013-7012 USA; 4 Department of Earth and Environmental Sciences, Mount Royal University, Calgary, AB T5E 6K6 Canada

**Keywords:** Ecology, Limnology

## Abstract

Magadiite, a rare hydrous sodium-silicate mineral [NaSi_7_O_13_(OH)_3_·4(H_2_O)], was discovered about 50 years ago in sediments around Lake Magadi, a hypersaline alkaline lake fed by hot springs in the semi-arid southern Kenya Rift Valley. Today this harsh lacustrine environment excludes most organisms except microbial extremophiles, a few invertebrates (mostly insects), highly adapted fish (*Alcolapia* sp.), and birds including flamingos. Burrows discovered in outcrops of the High Magadi Beds (~25–9 ka) that predate the modern saline (trona) pan show that beetles and other invertebrates inhabit this extreme environment when conditions become more favourable. Burrows (cm-scale) preserved in magadiite in the High Magadi Beds are filled with mud, silt and sand from overlying sediments. Their stratigraphic context reveals upward-shallowing cycles from mud to interlaminated mud-magadiite to magadiite in dm-scale units. The burrows were formed when the lake floor became fresher and oxygenated, after a period when magadiite precipitated in shallow saline waters. The burrows, probably produced by beetles, show that trace fossils can provide evidence for short-term (possibly years to decades) changes in the contemporary environment that might not otherwise be recognised or preserved physically or chemically in the sediment record.

## Introduction

Saline lakes precipitating evaporites are among the most extreme environments on Earth and include the most important stressors for metazoan life^1–3^. Hypersalinity is commonly accompanied by high pH (hyperalkalinity), anoxia and high turbidity, all of which are major stressors in underfilled evaporitic lakes^4–7^. Although metazoan diversity is typically low in these harsh settings, microbial communities often flourish^8,9^. Soda lakes frequently have very high microbial biomass (mainly photoautotrophs) and productivity^10,11^. Such lakes, with their carbonate-rich waters, are most common in regions with volcanic bedrock or hydrothermal recharge^12,13^.

Lake Magadi, a hypersaline (>300 g/kg TDS: Total Dissolved Solids) alkaline (pH: 10–11) soda lake, 0–2 m deep, lies in faulted volcanic terrain in the axial depression of the southern Kenya Rift^14–16^ just south of the equator (1°53′S). Modern lake sediments, cores and Quaternary deposits exposed around its margins have provided details of the sedimentary facies, including thick deposits of trona [Na_3_(CO_3_)(HCO_3_)·2H_2_O] that underlie the modern lake floor^14,16,17^. The Pleistocene sediment record includes abundant bedded, nodular and intrusive chert (including dykes) of diverse origins and the rare sodium-silicate mineral, magadiite [NaSi_7_O_13_(OH)_3_·4(H_2_O)]^18–23^. Lake Magadi, the most saline of the major lakes in the East African Rift, is one of the most chemically stressful lacustrine settings on the planet^24^. In this paper, we describe and interpret unusual trace fossils that are preserved in terminal Pleistocene magadiite. This soft silicate mineral, a precursor of quartzose chert, is an improbable host for invertebrate ichnofossils. The trace fossils, nonetheless, provide new clues to the origin and history of sedimentation of bedded sodium-silicate minerals in saline alkaline lakes.

## Geological Setting of the Trace Fossils

**Figure 1:**
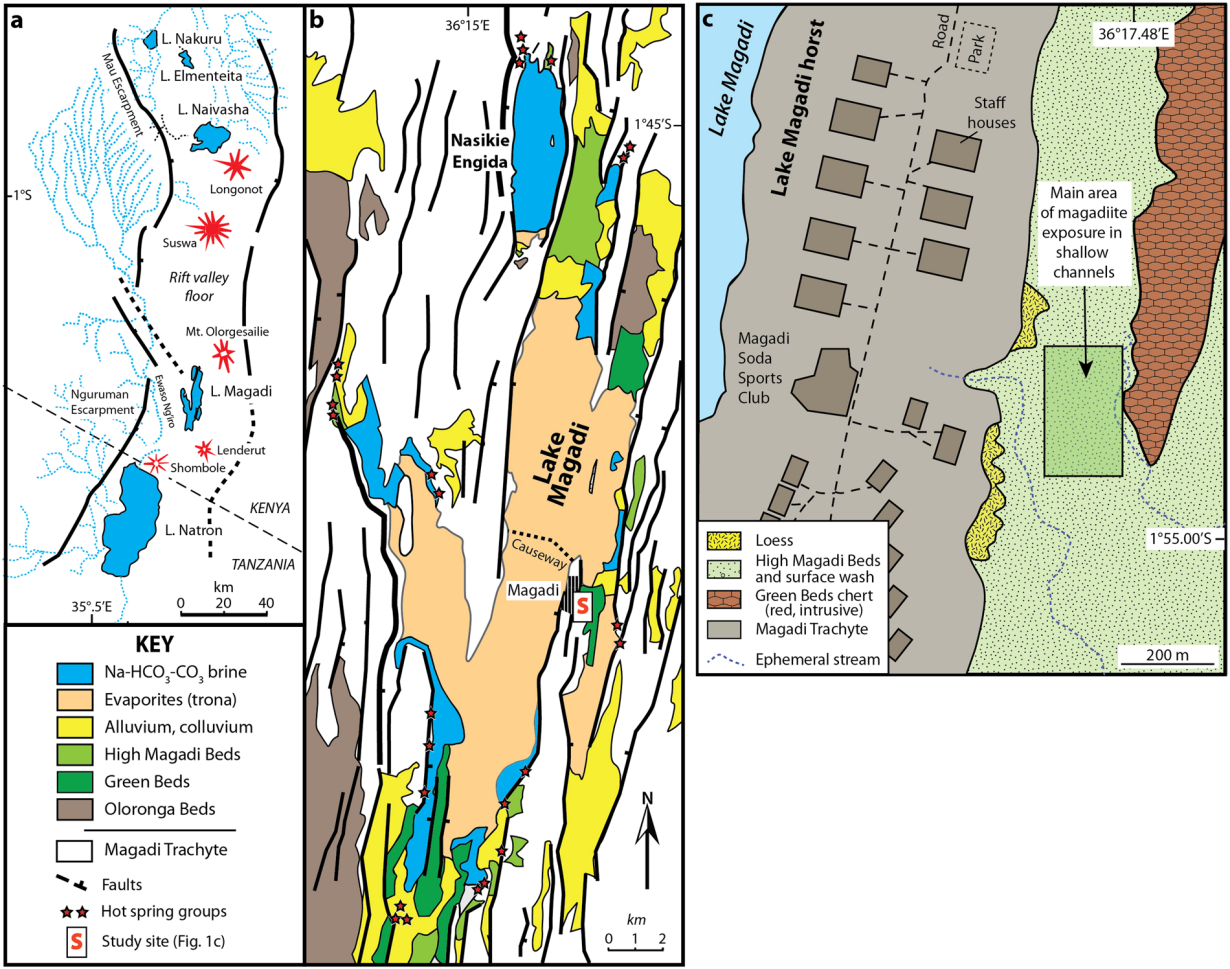
Geology of Magadi basin showing the study site. (**a)** Location of Lake Magadi in the southern Kenya Rift. (**b)** Simplified geological map of the area around Lake Magadi. For details of local geology see refs. ^14,27^ (and references therein). ***S*** shows location of the study site with the trace fossils. (**c)** Local details of the location (***S***) where the trace fossils were found. Maps produced by RWR using Adobe Illustrator CC v. 23.0.4.

Lake Magadi lies in a faulted terrain of Pleistocene volcanic rocks in the axial trough of the southern Kenya Rift at ~605 m above sea level^14,16^ (Fig. 1). The lake, which is fed by saline hot springs and seasonal runoff^15,25^, lies in a region with a strongly negative precipitation/evaporation budget. The stratigraphic succession, exposed discontinuously around its margins, consists of the early to mid-Pleistocene Oloronga Beds (fluvial and lacustrine), which lie upon Magadi Trachyte (1.4–0.8 Ma) basement, and are locally overlain by calcrete^26^, the lacustrine Green Beds and associated intrusive chert (191–158 ka), the High Magadi Beds (HMB: ~25–9 ka: mainly lacustrine), and the Evaporite Series (<9 ka to present: lacustrine evaporites and organic muds)^17,21,27^. Sedimentary rocks younger than the Oloronga Beds are confined mainly to the axial N-S graben.

**Figure 2:**
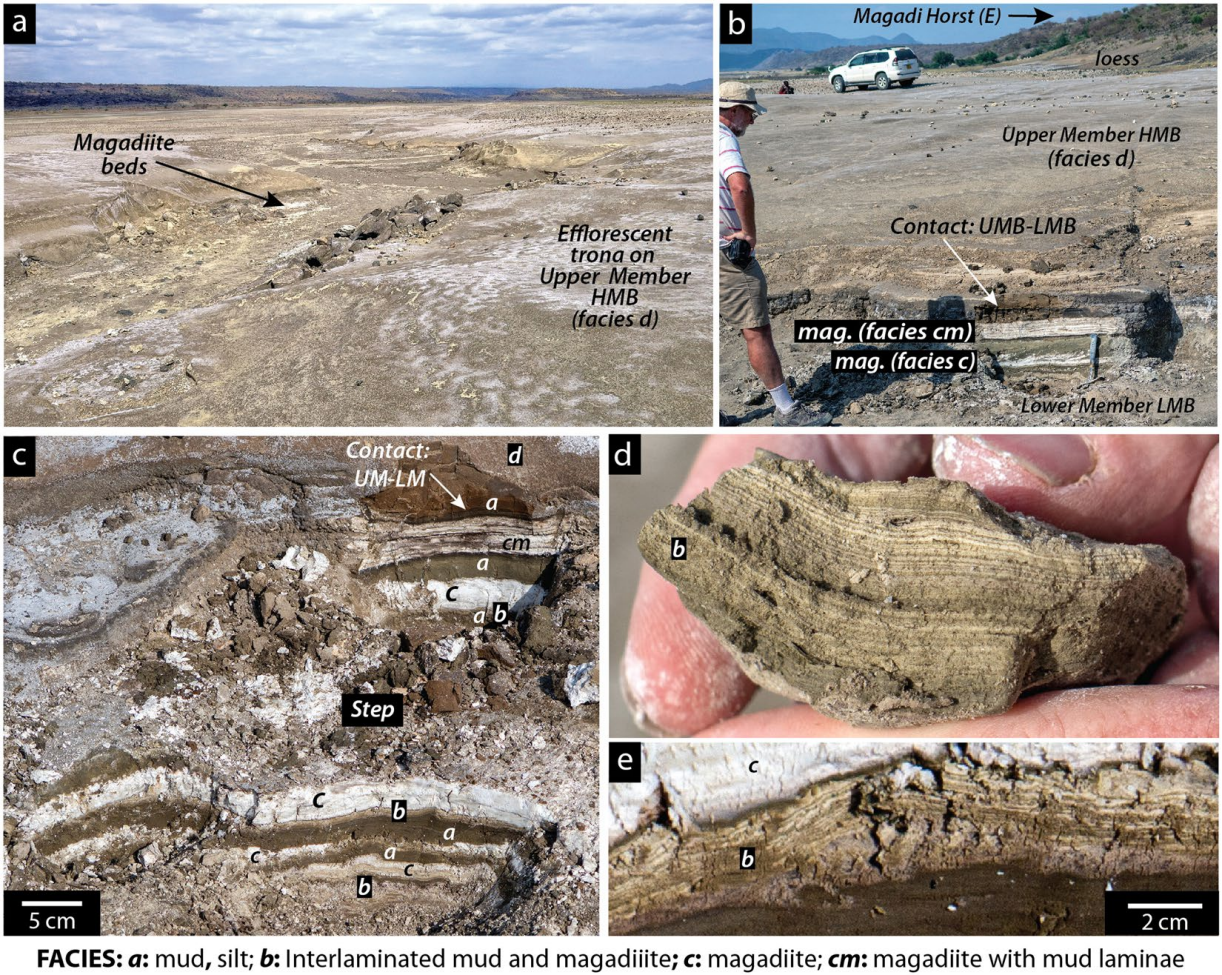
Location of the traces in outcrop. (**a)** View southwards of the eastern sub-basin showing High Magadi Beds in shallow ephemeral-stream channels. Exposed magadiite beds are white to grey in outcrop and commonly encrusted by efflorescent trona up to 1 cm thick. UM HMB: ‘Upper Member’, High Magadi Beds (facies ***d***). The land surface is mainly volcanic silts of the upper unit (UM) of the High Magadi Beds (HMB). (**b)** Shallow pit exposing two magadiite beds (m: facies ***c***) and the contact between the ‘Upper Member’ (UM) and ‘Lower Member’ (LM). (**c)** Stepped trench exposing two magadiite beds shown in B, but ~50 m to the south. Letters in italics (***a***–***d***) refer to the facies described in the text. (**d)** Facies ***b*** showing regular, alternating fine laminae of magadiite and siliciclastic mud. (**e)** Facies ***b*** showing laminae disrupted by cracks and other irregularities, overlain by magadiite (facies ***c***) with a dark (organic?) lamina at the contact. Photographs taken by RWR.

Burrows were discovered in bedded magadiite deposits of the High Magadi Beds, a series of fluvial, lacustrine and volcaniclastic sediments up to ~6 m thick in outcrop, exposed up to ~12 m above modern Lake Magadi, that record former high lake-levels of terminal Pleistocene to early Holocene age in the Magadi Basin^14,16–19,27,28^. The outcrop with ichnofossils lies at ~605 m elevation, ~100 m east of the base of the narrow (<1.5 km wide) N-S-trending Magadi horst that separates the southeastern sub-basin from the axial trona pan (Figs. 1c, 2). The horst is composed of Magadi Trachyte, which underlies much of the southern Kenya Rift floor^14,29–31^. Greenish grey and red chert dykes lie along the base of the fault escarpments bordering the northeastern and northwestern edges of the Magadi horst^19,21,32^. East and southwest of the horst, coalescent domal mounds of massive, brecciated and laminated reddish or pale green chert up to 1.5 m thick are present^27^, with some <150 m east of the study site (Fig. 1c). The High Magadi Beds disconformably overlie these Pleistocene cherts, which belong to the Pleistocene Green Beds^21^.

The section with trace-fossil-bearing magadiite crops out in a small ephemeral N-S channel 3–8 m wide and up to 1 m deep with low-angled margins, and its minor W-E tributaries (Fig. 2a,b). The magadiite bed containing burrows, which is ~10 cm thick, overlies interlaminated magadiite and silt, and is overlain by greenish brown silt and mud (Fig. 2c,d). The darker silt and fine-grained sand (Facies ***a***) provides the lithological contrast that highlights the burrows in magadiite. That silt in turn is overlain by interlaminated magadiite and mud (Facies ***b***), another magadiite bed (Facies ***c***), dark brown mud, and thicker beds of light brown, weakly bedded to laminated volcaniclastic siltstone (Facies ***d***; Fig. 2b,c). A lower unit (“lower member”) of lacustrine sediments, and an upper unit (up to ~5 m thick, “upper member”) represented by pale brown, volcaniclastic siltstone were recognised in a survey of High Magadi Beds outcrops throughout the Magadi Basin^16,19^. The trace fossils are present in the uppermost part of the lower member, the base of which is not exposed near the outcrops in the southeastern sub-basin. Thicker units of High Magadi Beds are present in cores recovered from the lake floor^14,17,27,33–36^. The informal divisions of the HMB based on outcrop will likely be revised.

X-ray diffraction (XRD) analyses show that the weakly lithified silt and mud above and below the several magadiite beds in outcrop are composed of the authigenic zeolite erionite [(Na_2_,K_2_,Ca)_2_Al_4_Si_14_O_36_·15H_2_O], detrital K-feldspar (mainly anorthoclase) and amphibole, with variable amounts of authigenic analcime and quartz^33,37^. Calcite is a common late precipitate in cracks and secondary pores. Silt and mud above the uppermost magadiite bed also contains fine plant debris, dark brown organic matter and thin (<5 mm) layers with fragile black fish-bones (*Tilapia* or *Oreochromis* sp.), many of which are poorly preserved.

### Trace fossil description and interpretation

**Figure 3:**
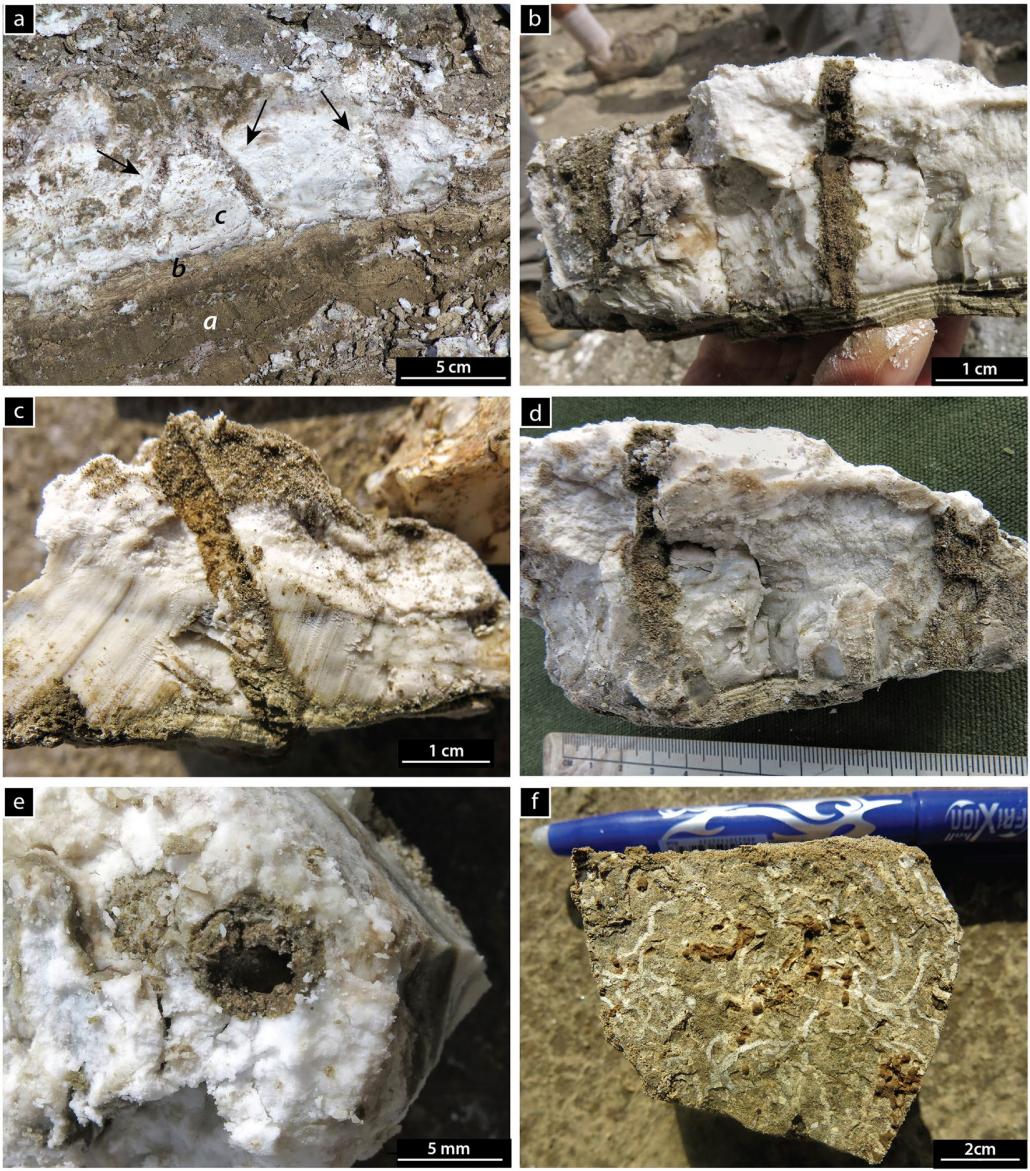
Trace fossils in outcrop. (**a)** Large burrows (arrows) in bedded magadiite **(**facies ***c*)**. Underlying facies ***a*** and ***b*** are also shown. **(b–d**) Close-up cross-section views of large, vertical to inclined burrows penetrating into the magadiite bed from an overlying colonization surface. (**e**) Bedding-plane view showing burrow opening. (**f**) Bedding-plane view of small horizontal burrows. Photographs taken by LAB.

The trace fossils are preserved in full relief (Fig. 3). They consist of simple cylindrical, unbranched, mostly unlined burrows with sharp margins, filled with sediment different from the host substrate. At the contact between the magadiite bed and the overlying silt, the burrows are horizontal, but are oblique to vertical in the magadiite bed. Some burrows, however, become horizontal again ~2 cm below the overlying magadiite-silt contact. Burrow fill consists of structureless, dark grey, reddish brown and black silt and fine-grained sand derived from the overlying sediments, which contrasts strongly with the white to pale grey magadiite host. Two size-populations were identified. Large burrows, 4.7–7.0 mm wide, penetrate the magadiite up to 51.6 mm below its upper contact (Fig. 3b–e). A clayey lining may be present, with very fine-grained sand fill of sub-mm aggregates and structureless backfill (Fig. 3b). The smaller burrows are 1.4–1.5 mm wide and up to 23.4 mm deep (Fig. 3f).

The trace fossils in magadiite resemble a few ichnogenera described from the fossil record. Simple, unlined burrows with a fill that contrasts with the host rock, and typically a dominantly horizontal orientation, are usually assigned to *Planolites*. This ichnogenus, however, is usually interpreted as being produced by a deposit feeder that actively filled its burrow. The fact that the magadiite burrows are filled with sediment from the overlying sediment layer implies passive fill and (or) active fill downwards into the magadiite horizon. Attribution to *Planolites* would therefore be unwarranted. In contrast, *Palaeophycus* is passively filled but, unlike the magadiite trace fossils, has a lining. Some of the larger trace fossils do preserve a clay lining and very fine-grained sand fill, which appears to be at least partly backfilled with sub-mm sediment aggregates in the vertical portion of the burrow (Fig. 3b). Accordingly, we prefer to leave these unusual burrows in open nomenclature.

Given the paucity of modern bioturbators in soda lakes, few candidates are considered as potential tracemakers, but we recognise that different environmental conditions might have existed soon after the magadiite bed formed. Two species of beetles^38,39^ and one species of chironomid^24^ have been documented in Lake Magadi in biological literature, and more invertebrates were observed in modern lake-margin environments during 2007 and 2008^40,41^. The water scavenger beetle *Coelostoma tina* (Hydrophilidae: Sphaeridiinae) lives in lagoons and pools surrounding modern Lake Magadi^39^. Hydrophyloid beetles are extremely diverse^42^. Most hydrophyloids are aquatic and some swim well^42,43^. Although most Sphaeridiinae are terrestrial, they often inhabit areas with a high water-table; *Coelostoma*, in particular, is aquatic^43^. Hydrophyloid beetles produce traces, but those records are poorly documented^44^. The size of *Coelostoma tina* is consistent with the size of the larger burrows recorded. The aquatic habitat of these beetles makes them potential producers of the magadiite burrows, given that most sedimentological evidence implies that colonization of the magadiite substrate occurred in shallow water (see Discussion).

The chironomid (lake fly) *Tanytarsus minutipalpus* lives in modern Lake Magadi^24^. Chironomids commonly inhabit stressed settings, including hypersaline and oxygen-depleted lakes^45–47^. Chironomid larvae produce burrows, including simple U-shaped and branched systems in many lake types^48–51^. Burrow systems attributed to chironomids have been documented elsewhere in the Kenya Rift Valley^52^. The size of *Tanytarsus minutipalpus* is consistent with the dimensions of the smaller burrows studied. Their aquatic habitat adds support for chironomid larvae being possible producers of the smaller burrows in the magadiite. However, the morphology of the burrows documented in this study seems to be simpler than those typically attributed to chironomids.

The tiger beetle *Lophyra pseudodistans* (Carabidae: Cincindelinae) is endemic to the Lake Magadi area^38^. Tiger beetle larvae produce vertical burrows similar to those recorded in the magadiite^44,53,54^. The air-breathing habit of tiger beetles is inconsistent with sedimentological evidence that implies aquatic conditions during colonization, although the fill of some of the burrows is coarser-grained mud pellets that might indicate a period of non-deposition following shallowing and temporary exposure of the substrate. Tiger beetle larvae, which typically inhabit wet marginal settings, can survive flooding of their host sediments from several hours up to months^54–57^.

Staphylinid beetles, like tiger beetles, produce burrows in the moist to saturated lake-marginal sediments around modern Lake Magadi and Lake Bogoria (Kenya)^40,41,58,59^. Their burrows, in contrast to tiger beetles, are both vertical to oblique with extensions to horizontal tunnels at the sediment-air-water interface^60–62^. Their vertical burrows may have narrow constrictions to a smaller opening at the sediment surface, which allows the beetles to survive flooding and periods of anoxia^63^. In semi-soft to firm cohesive microbially bound mud substrates known from Lake Magadi and Lake Bogoria, the burrow margins are sharp, the mix of backfill and passive-fill sediment differs from the host and is typically slightly coarser-grained than the host, as is also the case at Magadi^40,59^. The siliceous substrate at Magadi would presumably also have been similarly cohesive and semi-soft.

## Discussion

Saline alkaline (soda) lakes are extreme environments for most organisms. Although microbial extremophiles often flourish in these settings, few animals survive under the prolonged stressed conditions that characterise these lakes^7,24,47^. Hydrologically closed lakes nonetheless are dynamic environments that can undergo rapid, and sometimes extreme, changes in salinity and alkalinity during periods of freshening or evaporation that are usually tied to increased dilute inflow from the drainage basin (rivers and direct rainfall), varying recharge from groundwater, or loss of inflow during arid periods^64^. Such changes are often linked to climate variations on annual to decadal to centennial or millennial timescales, but also to longer-term tectonic modifications of the hydrology (e.g., river diversions) and hydrogeology in the evolving rift^65,66^.

Hypersaline lakes can become brackish or even dilute within a few years of freshwater inflow. Lake Bogoria, for example, a perennial highly saline (>50 g/L TDS in summer 2006) lake in the central Kenya Rift, became fresh enough to support crocodiles, fish and hippopotamuses a few years later after prolonged heavy rains^67^. In saline lakes, the biological diversity often increases initially near fluctuating lake margins where local littoral and shallow-offshore freshening accompanies rising lake level, while low diversity persists in deeper parts of the lake because of ponding of dense saline water in lake-floor depressions or chemical stratification in the water column^68^. Together these often lead to anoxia in the hypolimnion or monimolimnion. In contrast, carbonate and evaporite precipitation typifies falling and lowstand conditions in shallow ephemeral saline lakes^4^. Saline pans and residual pools, often near spring-fed shrinking evaporitic lakes, are stressful environments that typically preserve few biogenic structures^2^. Hypersalinity and oxygen-depletion can result from water stratification during oligohaline (brackish) phases (e.g., flooding by fluvial inflow), increasing stress on benthic organisms. The presence of macroburrows in magadiite, which precipitated in the most saline of the large modern lakes in the East African Rift, is therefore surprising. A critical issue is the chemical environment of the submerged or briefly exposed lake floor during burrowing.

Magadiite, first described at Lake Magadi^18–20,33^, is the most common of several rare sodium-silicate minerals (including kenyaite, makatite and kanemite) that form in saline, highly alkaline lakes^69–73^. Magadiite at Lake Magadi is present in brown and dark green lacustrine muds as irregular nodules, up to ~15 cm long and 5 cm thick, thin (<5 mm) lenses and dispersed patches (mm–cm scale), and as thin laterally-continuous laminae (<3 mm) and beds (<15 cm) interlayered with zeolitic lacustrine silt and mud, some rich in dark brown to black organic matter^18,19^.

Chemical conditions for magadiite formation are well known, and the mineral can be synthesized in the laboratory^74–78^. It has been proposed that bedded magadiite interlayered with muddy sediments formed at the chemocline of a stratified lake (intermittent stratification or meromixis)^16,18–20^. In that model, the mineral precipitated where evaporated highly alkaline, silica-rich sodic brine became overlain by more dilute, less dense water, as for example during seasonal flooding by runoff. The lowered pH at the fluid interface would decrease silica solubility in the underlying brine^16–20^. Magadiite forming in the water column would then settle gravitationally on the lake floor, forming a soft, possibly gelatinous layer composed of fine spherulitic grains^21^. In a stratified water column, the different densities of the water masses would limit wind mixing or overturn until evaporation of the surface waters (epilimnion or mixolimnion) led to approximately equal densities. For pure magadiite to form implies that little detrital sediment was then present in the surface waters. Lowering the pH in a sodic brine could also have been induced by biogenic CO_2_ released from decay of degrading organic matter^19^, or possibly from pulses of CO_2_ of geothermal (upper mantle to lower crustal) origin^79^. One cm of magadiite is thought to form in about 20 years^19^.

Modern examples of magadiite precipitation are rare, so the physical state of magadiite soon after precipitation is unclear. However, recent magadiite formation has been documented in a core from Lake Kitagata (Uganda), a small shallow (9 m deep) perennial saline (>170 g/LTDS) alkaline (pH 9.7) crater lake fed mainly by groundwater^80^. There, the magadiite forms in the silica-rich (256 ppm SiO_2_) water column as a primary mineral that settles upon the substrate and in places forms thin beds. Some magadiite grains contain muddy inclusions implying some *in situ* formation by intrasediment growth. The lake is meromictic with a chemocline at 1 m depth^81^.

In contrast, the formation of magadiite in an evaporative brine pond has been reported at Alkali Lake, Oregon, USA^82^. That pond was fed partly by dilute alkaline well-water. The authors reported “ball-like aggregates of white semi-crystalline material with patchy opalescence”. X-ray diffraction analyses confirmed magadiite and clay minerals. Magadiite might also precipitate directly from solution by evaporative concentration^20^, as is the case of littoral muddy plains at Lake Magadi that were temporarily covered with shallow brine undergoing evaporation^21^. The thickest magadiite unit at the base of the Lake Kitagata core is crumbly, overlies a palaeosol, and probably formed by capillary evaporation when the crater lake had almost desiccated^81^. Magadiite has also been reported from Late Pleistocene and recent sediments in Lake Bogoria^64,72,83,84^, Lake Chad^70,71,73^, Manga in Niger^71^, Sua Pan in Botswana^85^, and Malha crater lake in Sudan^86^.

The ichnologic evidence provides clues to the magadiite origin at Lake Magadi. Whether magadiite precipitated by evaporation of a shallow, silica-rich sodium carbonate brine or in a deeper chemically stratified alkaline lake, the lake-floor environment during colonization – benthic or temporarily subaerial – would initially have been hostile to most organisms except bacteria and archaea. The contact between the trace-bearing magadiite bed and the overlying lacustrine silt and mud is thus regarded as the primary colonization surface, although some burrows penetrated the magadiite from up to ~8 cm above the contact. In some examples, the burrow fill is coarser-grained than the overlying silt or mud, and represents a period of time on the colonization surface where these sediments were locally available before continued mud deposition. Accordingly, environmental conditions during colonization were either those associated with the initial silt or mud deposition upon the underlying magadiite substrate, or from this colonization surface during a short depositional hiatus.

**Figure 4:**
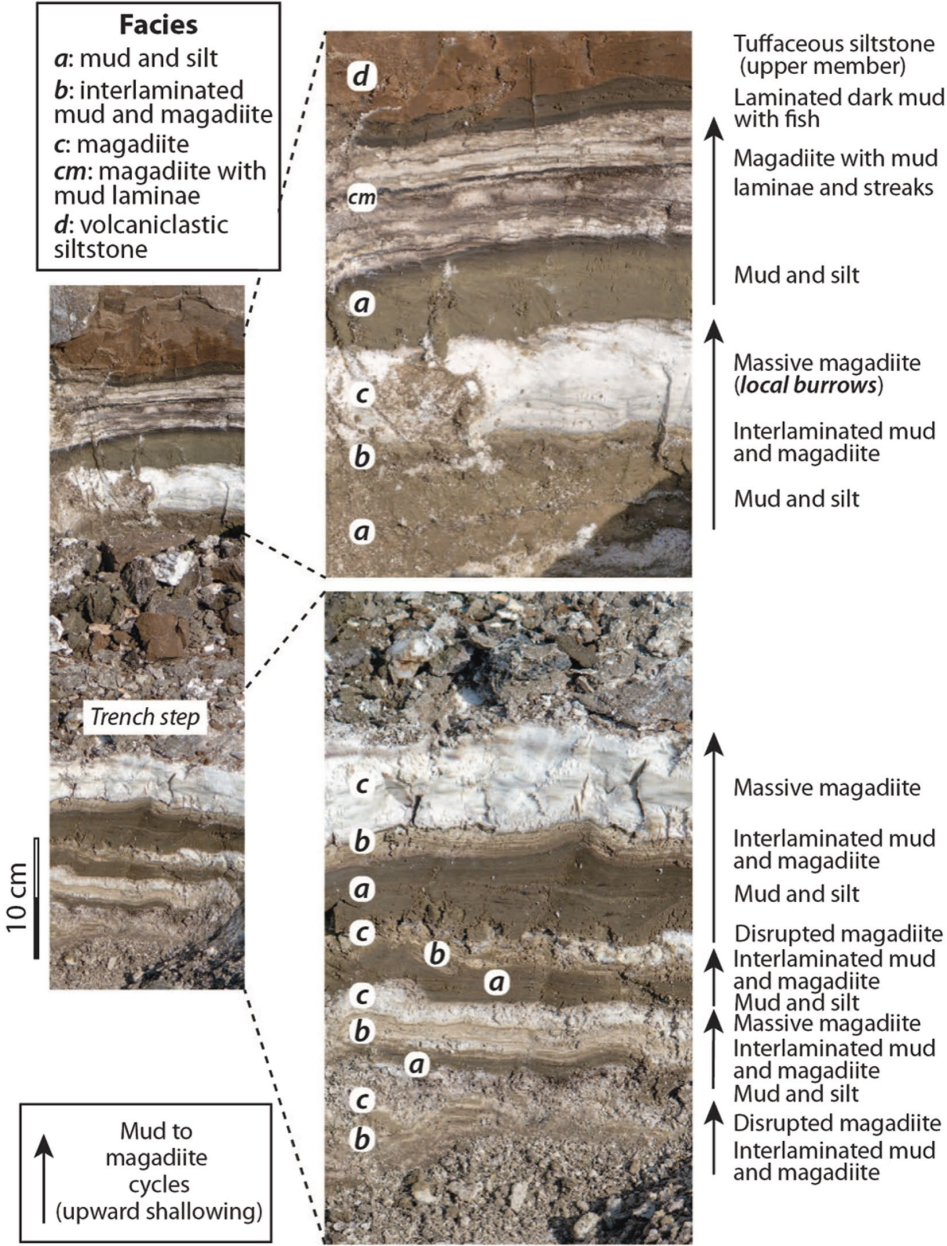
Field photographs that show repeating (cyclic) patterns in sedimentation at the study site. Each arrow indicates one shallowing-upward cycle. Facies codes ***a***–***d*** are described in the text. Photographs taken by RWR.

The exposed sediments with distinct magadiite beds, including the one containing burrows, show repeating facies successions (‘cycles’) on a cm–dm scale that give clues to the pattern of sedimentation when the ichnofossils were formed (Fig. 4). We interpret each cycle to begin with lacustrine silt and mud and terminate with bedded magadiite of variable purity and thickness. Each magadiite bed is overlain by dark to greenish brown zeolitic silt, fine sand or mud (Facies ***a***), some of which shows indistinct lamination. The contact, interpreted as a small-scale flooding surface, is usually sharp, but locally irregular because of minor compaction of muds into the underlying soft magadiite, local W-E slippage from the Magadi horst, or local erosion. These silt, fine sand and mud layers, in turn, are commonly succeeded by a few cm of interlaminated mud and magadiite (Facies ***b***), which is commonly undulating or wavy in sectional view (Fig. 2c). Those laminae are continuous in some units (Fig. 2d) but broken or disrupted with small-scale disconformities in others (Fig. 2e). Where undisturbed, the laminae lie parallel to laminae in the underlying brown mud and silt if present, and commonly inherit underlying morphological irregularities in the substrate. These laminated units are then capped either by white or grey magadiite (Facies ***c***) or magadiite containing thin brown mud laminae (Facies ***cm***), typically with sharp upper and lower contacts (Fig. 4). The thickest magadiite beds have fewest impurities. Some are streaky in outcrop with eastward-dipping magadiite layers thinning westwards from the horst (Fig. 2b).

**Figure 5:**
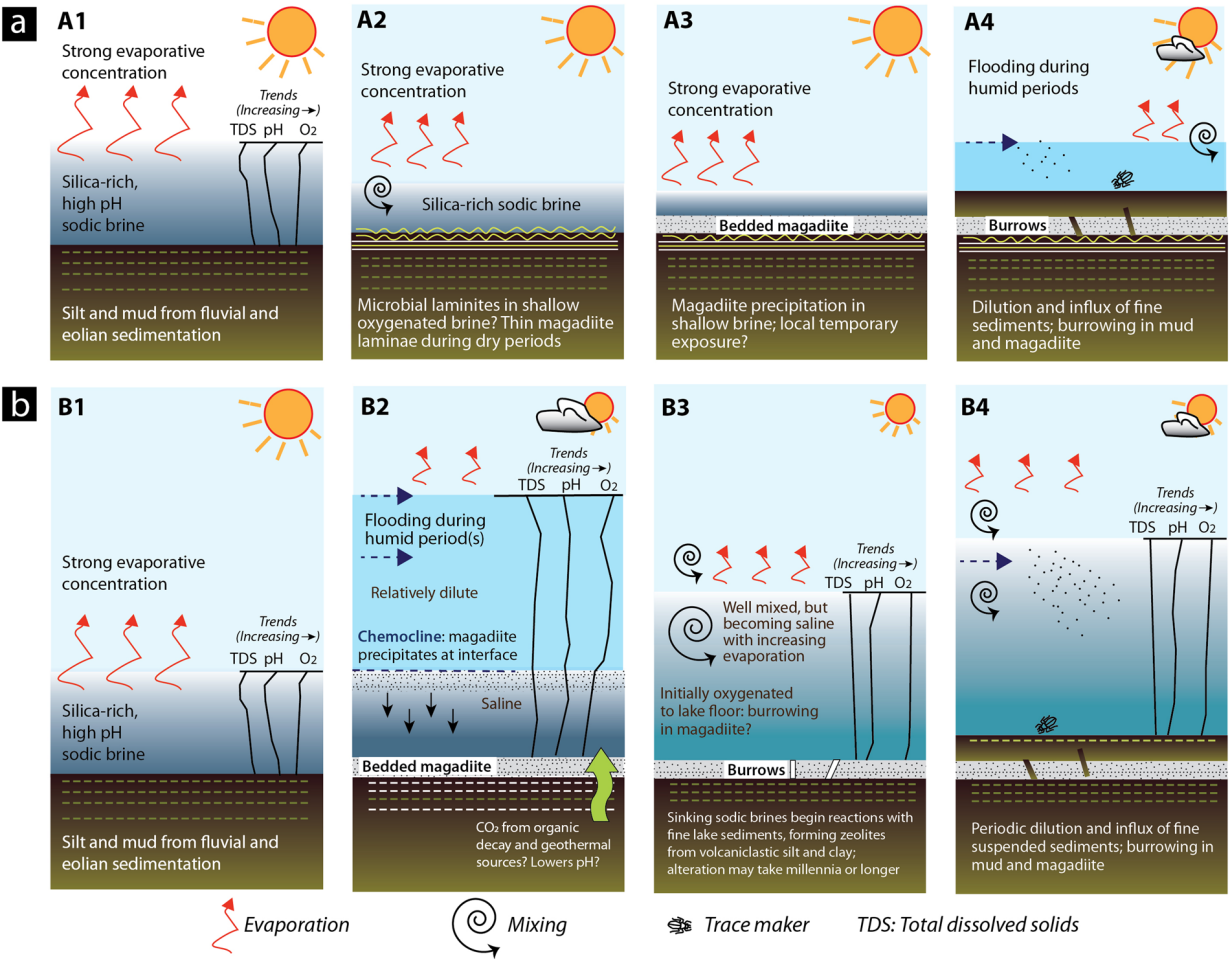
Models for the origin of bedded magadiite and its trace fossils. **(a) A1** to **A4** show the *shallow-water model*, which is best supported by our evidence. **A1**. Following deposition of fine clastics, lake water evaporates producing an alkaline sodic brine with high pH (>10). High aqueous silica concentration. **A2**. The brine attains saturation with respect to magadiite which precipitates seasonally, alternating with siliciclastic mud in shallow (<1–5 m?) water to form mm-scale laminites. Undisturbed laminae imply bottom-water anoxia. Where the lake floor is shallower and at least temporarily oxygenated, microbial mats might influence or control benthic sedimentation. **A3**. With continued evaporation the shallowing brine becomes supersaturated with respect to magadiite, which precipitates and settles rapidly as soft gelatinous material that extends laterally for hundreds of metres and forms thinly bedded magadiite on the lake floor. Some beds become contaminated by siliciclastic sediments derived from ephemeral-stream inflow (facies ***cm***) and eolian dust (or volcanic ash). In lake marginal settings and periods when lake level is unusually low, some shallow magadiite might become subaerially exposed. This provides an opportunity for organisms (subaerial and shallow subaqueous: aerobic or dysaerobic that need oxygen) to traverse the littoral zones and feed on newly exposed microbial organic matter. **A4**. Rise in lake level delivers fine siliciclastics and provides fresher water. Organisms adapted to fresher waters burrow into underlying siliciclastic sediments and magadiite. Some open burrows become filled by fine sediments or remain open for minerals precipitated during later evaporation. **(b) B1** to **B4** show the *perennial lake model* in which magadiite precipitates at the interface of a stratified lake, when shallower dilute lake waters move across a denser sodic brine. After deposition of fine clastics at the interface, lake water evaporates producing a sodic brine with high pH. Aqueous silica concentration remains very high. **B2**. Flooding linked to increased rainfall (or drainage diversion?) dilutes Lake Magadi and a stratified lake develops. Magadiite then precipitates at the chemocline. Initially the magadiite may alternate with mud, forming laminites. **B3**. With evaporation and shallowing the waters become mixed and oxygenated to the lake floor. Burrowing occurs while conditions remain favourable. **B4**. Flooding introduces fresher water and sediment. Evaporation gradually increases the salinity. Drawing produced by RWR using Adobe Illustrator CC v. 23.0.4.

The critical question in interpreting these cycles is whether the magadiite bed(s) records precipitation in a shallow lake where magadiite formed by (1) evaporative concentration of a silica-rich sodic brine in shallow oxygenated water, or in (2) a deeper stratified lake with precipitation at a chemocline (Fig. 5). The burrows imply at least partial or temporary oxygenation of the lake floor when they formed, and (or) a brief period of exposure before continued sedimentation. That in turn has implications for understanding the origin of bedded magadiite.

The cycles (Facies ***a*** to ***c***) are interpreted as shallowing-upward successions in shallow (dm to a few metres?) water (Fig. 5 A1–A4). The magadiite beds, which represent the most saline part of the cycle with high Na^+^ and aqueous SiO_2_, are periodically flooded by runoff that introduces detrital fine sand, silt and clay that are deposited upon a magadiite substrate. The aqueous environment during flooding was probably oxidizing, but initially brackish given that the inflow water flooded saline water and (or) a saline substrate. Mud-silt sedimentation continued from seasonal runoff but as the water shallowed and increased in salinity, it became sufficiently saline for magadiite precipitation, which for a period alternated with thin mud laminae. Some alternations are regular (Fig. 2d), implying that they could have been seasonal with magadiite precipitating in the drier periods and mud settling gravitationally during the rainy seasons. This speculation is unconfirmed. These couplets, however, resemble microbial laminites in lacustrine upward-shallowing cycles. Most laminae are parallel and unfractured, implying that they formed and were preserved in shallow water. Some that are broken or crinkled may record brief periods of subaerial exposure or near exposure (Fig. 2e). A darker layer <1 cm thick that locally underlies the laminite zone (Fig. 4) could represent a former layer of bacterial reduction below organic laminites. A thin dark microbial (?) lamina is also present below some magadiite beds (Fig. 2e).

The magadiite beds that cap the cycles represent the most saline (Na- and silica-rich) fluids. The thickest and purest beds might have formed in shallow brine upon underlying subaqueous laminites. The thinner and more irregular magadiite layers could have been disrupted by intermittent subaerial exposure or intrasediment growth at a very shallow water table, but the overlying siliciclastic mud and silt shows no clear evidence of exposure. Were the thicker magadiite beds subaerially exposed? Where subaerially exposed today by erosion, soft plastic magadiite rapidly becomes encrusted with efflorescent trona, and soon forms a thin (1–3 mm) hard grey crust. The uppermost surface (bedding plane) initially develops small (2–4 mm wide) cracks on exposure and begins to transform to quartzose chert with reticulate crack networks during early diagenesis (see below). This was not observed at the study site because the bedded magadiite was buried until excavated but has been observed elsewhere at several locations in the southern Magadi basin. Contemporary development of a hard silica crust, resulting from dehydration during exposure, would have prevented, or at least inhibited, the tracemakers from burrowing in still soft, plastic magadiite. The sharp, locally irregular upper contact also implies that the magadiite remained submerged during the next flooding event that introduced siliciclastic mud. Magadiite intraclasts were not found in the overlying basal muds. The most likely setting for this scenario would have been in a shallow (<1 m deep at the study site?) lake and laterally adjacent mudflats.

The alternative explanation would be for magadiite precipitation in a stratified lake^16,19,20^. Cycles in a stratified lake would result from mud-silt deposition during relatively humid phases followed by evaporation in a lacustrine offshore setting (Fig. 5B1–B5). After precipitation of magadiite in a shallow silica-rich alkaline brine, inflow of freshwater bearing fine clastic sediment might lead to temporary stratification, with magadiite precipitating in the water column at the fluid interface, where the pH was lowered along the contact of the upper and lower fluids. Alternatively, when CO_2_ was being released from biogenic or deeper geothermal sources into the bottom waters (hypolimnion or monimolimnion). Clastic laminae would form during seasonal inflow of mud and silt from ephemeral streams similar to the lake-shallowing model, but without photosynthetic benthic microbial mats because of anoxicity below the fluid interface (i.e. chemocline). Those inflows would likely form hypopycnal plumes, as happens today at Lake Bogoria^87^. Evaporation would then lead to precipitation of thicker magadiite beds in the offshore brine until aqueous Si and Na^+^ declined in the surface waters. Further evaporation would lead to trona precipitation, but this is not evident in the exposed HMB sections. Decreasing pH, resulting from organic matter decay or geothermal inflow, would not necessarily be seasonal, making this a less likely option for producing repeating couplets observed at the study site and elsewhere. The ichnologic evidence is less consistent with this scenario because it implies that the producers were able to migrate deeper into the water body. Assuming that the producers might have had similar environmental range to the beetles in modern Lake Magadi, this scenario is unlikely, however, because the latter live either in very shallow pools (i.e. *Coelostoma tina*) or are essentially terrestrial (i.e. *Lophyra pseudodistans*)^38,39^. The lake floor at neighbouring Nasikie Engida, a small shallow (1.6 m deep) saline alkaline lake northwest of Lake Magadi (Fig. 1b), was anoxic at only 1 m water depth when measured^88^. Although tiger beetle larvae and staphylinids can survive periods of flooding and even anoxia^55,56,63^, they inhabit marginal areas that are often subaerially exposed. An offshore setting in a stratified lake would be flooded perennially over time periods much longer than those during which burrowers could survive.

After deposition of the magadiite beds capping the exposed lacustrine sediments (Fig. 4), the lake expanded with deposition of black and dark brown, locally organic-rich mud preserving fossil fish. This basinwide ‘event’ of unknown duration is recorded along the N-S basin axis because this fish bed is a marker horizon in outcrops north and south of Lake Magadi^19,27^, implying deeper stratified waters with an anoxic monimolimnion. This fish-bed marker was identified as far north as southeastern Nasikie Engida. Magadiite in black anoxic muds has also been recorded at Lake Bogoria^71,72,83,89^, showing that the stratified-lake model for bedded magadiite genesis is feasible but modern examples of bedded magadiite are unconfirmed. That model does not, however, concur with the sedimentological and ichnologic evidence for shallow waters and lake-floor oxygenation during sedimentation at the Magadi study site.

**Figure 6:**
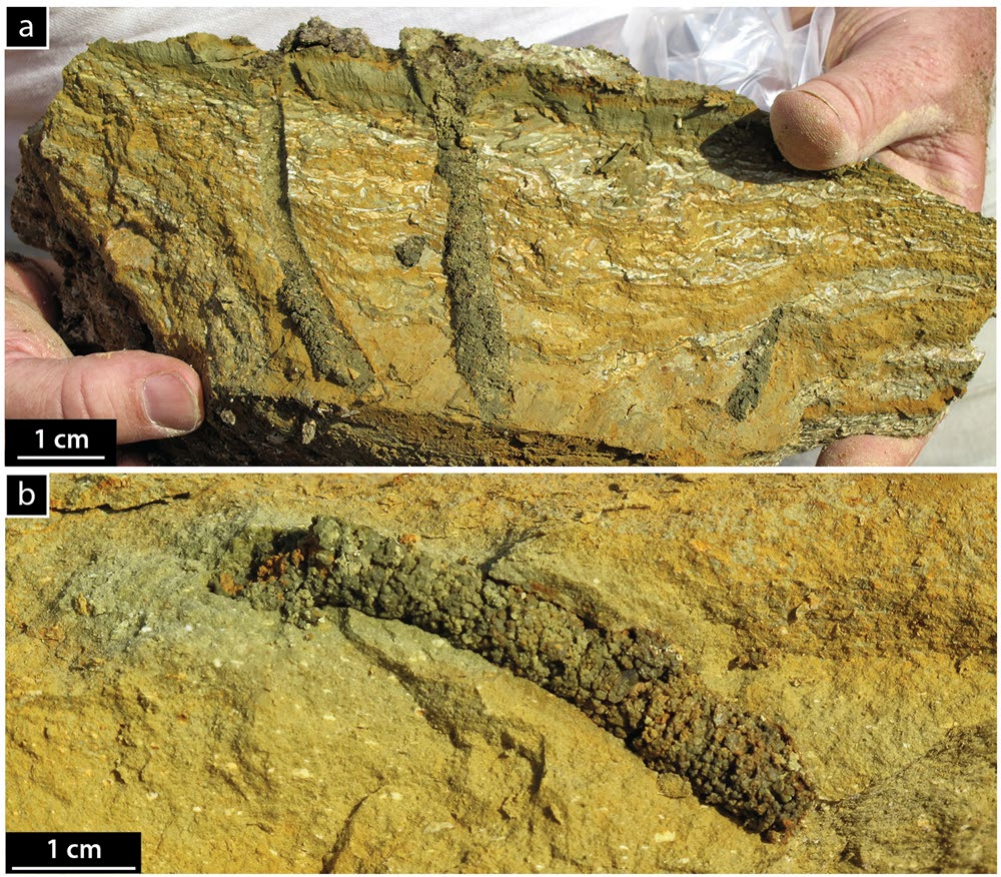
Burrows in the Pleistocene Green Beds from the southern end of Lake Magadi (see Fig. 5 in ref. ^27^) that are very similar to those in the HMB. **(a**) Burrows originate at the contact between lacustrine muds and an overlying darker layer of mud. The thin (1 mm) discontinuous white laminae below the contact are silica. Some of those patterns on bedding planes resemble those in Fig. 3F, but their nature remains uncertain. **(b)** Close-up of a single burrow showing infill contrasting with the host sediment. Photographs taken by RWR.

The Pleistocene Green Beds, which underlie the HMB disconformably, show a similar regressive succession in outcrops south of Lake Magadi^27^, where oxidized lacustrine silt and mud, some with large (cm-scale) burrows (Fig. 6), pass upwards into thinly bedded cherts in ‘cycles’ with a similar (cm-to-dm) scale to those of the HMB^40^. Bedded cherts in the Green Beds show possible stromatolitic layering, burrows, salt crystal pseudomorphs (calcite and Na-carbonates?), tepee and petee structures, and other features that imply shallow-water sedimentation on playa floors or upon broad littoral mudflats^19,21,22,32,41^. Most of those cherts, however, lack the characteristic reticulate crack-patterns commonly associated with diagenesis of a magadiite precursor^19,90^. Those patterns alone, however, are not necessarily diagnostic of former magadiite. The Green Beds chert precursor(s) might have been a siliceous gel (which form today at Nasikie Engida^91^, Fig. 1b), opaline silica, a carbonate mineral^21^, magadiite, or an as-yet-unidentified phase.

Magadiite, a metastable hydrous mineral, is a precursor of some lacustrine cherts. During early diagenesis, magadiite undergoes physical and chemical changes including loss of water and Na, and early recrystallization. Soft magadiite transforms progressively to microcrystalline quartz. Early diagenesis includes leaching of Na by percolating water followed by rapid recrystallization^18,19^. Using stable isotopes of oxygen at Olduvai (Tanzania), it has been shown that magadiite could ‘spontaneously’ recrystallize to quartz in saturated sodic brines^20,92^. This transformation sometimes involved intermediate phases including kenyaite [Na_2_Si_22_O_41_(OH)_8_•6(H_2_O)]^19^ and moganite (SiO_2_)^93^.

The timing of these diagenetic fluid-mineral reactions remains unclear but can be rapid (<10 ka and probably much less) because some magadiite in the High Magadi Beds has altered to kenyaite, moganite and chalcedonic microcrystalline quartz^18,19,33,93^. Early and rapid diagenesis also has implications for the interpretation of traces that were originally produced in semi-soft magadiite: first, their dimensions might change, reflecting shrinkage or expansion^52^ but early lithification could also reduce compaction. Second, uncemented siliciclastic fills might be physically flushed during or soon after lithification, especially with subaerial exposure in a closed lake with fluctuating level, leaving open pores or providing pore space for cementation by other minerals such as calcite or silica. The sharp burrow margins in the examples illustrated here indicate a semi-firm, but still moist, substrate.

Based on the evidence and discussion above, we infer that the fine sediments overlying the magadiite described in this study, although later altered to zeolites during early diagenesis^33,37^, record a period of more dilute lake water after the main phase of magadiite formation. During that time of generally fresher conditions, the trace makers inhabited the shallow lake floor, that for short times might have been subaerially exposed, and burrowed into the soft magadiite substrate. Continuing freshening is implied by the fossil fish upwards in the succession, notably in a dark, parallel-laminated layer approximately 10 cm above the top of the bioturbated magadiite bed. This cichlid-bearing layer has been radiocarbon dated at ~9,100 y BP^94–96^ placing it within the African Humid Period.

Chemically stratified lakes typically develop anoxic bottom-waters – a lacustrine environment unfavourable for macroscale trace-makers. Chironomids can tolerate extreme dysoxic conditions in lacustrine environments^45,50,97^. In contrast, most other burrowers, including water-scavenger beetles, which are possible producers of the large burrows at the study site, cannot survive extreme or prolonged dysoxia. Nevertheless, tiger beetle larvae, staphylinid beetles, and heterocerid beetles ― all known from hypersaline lake margins in the Kenya Rift ― are able to survive periods of flooding, and tiger beetles can even tolerate anoxic conditions during flooding for days to months^55,56,63^. In addition to freshening, increased dissolved oxygen might have developed on the lake floor. That would imply that the trace makers burrowed into an older magadiite bed rather than being contemporary with its precipitation, or they formed the burrows soon afterwards. Integration of ichnologic and sedimentological evidence implies that changing environmental conditions, whether freshening or brief subaerial exposure, might have favoured rapid colonization in a previously harsh benthic setting.

Lake water depth during magadiite sedimentation is unclear. A maximum elevation of 656 m for the HMB shoreline based on stromatolitic carbonates that extend southwards into Tanzania has been reported^98^ but remains unconfirmed by other sedimentological evidence^21,27^. A palaeoshoreline with stromatolite-coated gravel ~12 m above Lake Magadi along the fault scarp directly east of the Magadi horst^27^^,28^, and at many localities in the axial graben, provides clear evidence of a former HMB lake level that might have been contemporary with sedimentation at the study site. That lake would have been saline and alkaline during magadiite sedimentation and perhaps only a few metres deep at its maximum depth by analogy with other examples^64^ (e.g., Lake Bogoria^89^).

The laminated and muddy facies that overlie the magadiite beds record a change to deeper water above a more extensive flooding surface and by implication, more dilute and oxic conditions, at least in the waters that extended downwards to the lake floor during periods of burrowing. Interlaminated magadiite and muds, which overlie the burrowed magadiite bed, might indicate seasonal or irregular stratification of the water column with magadiite particles settling by gravity. The fossil fish record an expanded lake across much of the axial graben, implying deeper fresher water after a phase of more saline conditions.

Evaporitic soda lakes host simple food webs^7,47^. Modern Lake Magadi has a highly productive trophic web dominated by the heterotrophic bacteria *Arthrospira* sp., with detritus feeders and omnivorous chironomid larvae and copepods, the fish *Alcolapia grahami*, and several birds, including lesser flamingos, as primary consumers^7,24,47^. The addition of large burrowing insects to the aquatic benthos, most likely scavenging or predator beetles, would imply added complexity to the Lake Magadi food-web during periods of decreasing hydrochemical stress. Staphylinid beetles, however, are also primary consumers that feed on microbe-rich sediments at the sediment-air-water interface, and are well known from hypersaline lacustrine settings^61,99^. They can also survive inundation within their bottle-neck burrows, feeding on microbes during subsequent brief exposure-events^60,63^.

Although integration of ichnologic and sedimentological evidence is common practice in facies analysis, this process implies linking datasets that typically have marked differences in terms of temporal resolution and implications. Benthic colonization by burrowers may reflect short-term changes in environmental conditions that do not leave a physical or chemical record in stratigraphic successions. In this example, colonization in magadiite might have resulted from freshening and oxygenation of a shallow lake bottom and (or) brief exposure of the siliceous substrate. The bioturbation is decoupled from magadiite formation in terms of physical and chemical conditions. Detailed ichnologic analysis provides the clues for changing environmental conditions that are otherwise unrecorded or unrecognised in the sediment record.

## Conclusions

Burrows, probably produced by beetles, have been recorded in magadiite from the High Magadi Beds (~25–9 ka) in hypersaline, alkaline Lake Magadi in the southern Kenya Rift. This occurrence is unusual because the extreme environmental conditions in this lake are inhospitable for invertebrates. Based on sedimentological and fine-scale stratigraphic evidence, we conclude that the burrows were formed when the lake floor became temporarily fresher and oxygenated, after a period when magadiite precipitated in shallow saline waters, probably by evapoconcentration. Brief subaerial exposure is possible. Ichnologic evidence therefore provides palaeoenvironmental insights into short-term (possibly years to decades) changes that otherwise might have remained undetected when using physical or geochemical datasets alone.

## Materials and Methods

Bed-by-bed sedimentological analysis was performed in the field in 2015 following extensive fieldwork to determine the regional stratigraphy and sedimentology^27^. Trace fossils were analysed *in situ* and in the laboratory, using conventional practice^100^, which involves measurement and documentation of pertinent ichnologic features, such as burrow morphology, wall and lining, and trace orientation with respect to bedding. Preservation and inferred ethology were taken into account. Modern analogues for the traces and trace makers in lake-marginal settings were studied during 2007 and 2008^40,41^. Samples for X-ray diffraction analysis were analysed using a Panalytical X’pert Pro MPD diffractometer using CuKα radiation at 45 kV and 40 mA, respectively. Samples were prepared as pressed powder mounts after hand grinding with pestle and mortar, and as smear mounts on glass slides.
